# Where and How to Give

**Published:** 1903-05-16

**Authors:** 


### The Hospital.

A Journal of

The Medical Sciences and Hospital Administration.

Edited by Sir Henry Burdett, K.C.B.

Vol. XXXIV.—No. 868. [May 16, 1903.

### Where and How to Give.

The evils of indiscriminating charity have been dwelt on times without number by those who are in a position to judge of its effects, but their warnings for the most part have had reference to direct dealings between persons of charitable instinct, and those applicants for personal relief concerning whom they have no information beyond what the applicants are willing to supply themselves. The same sort of caution, however, might be exercised with great advantage to the general cause of charity in London when considering applications for support from any hospital or charitable institution with the working of which donors have no intimate personal acquaintance. The sympathies of the public are very easily aroused by the urgent appeals which the managers of some hospitals and charitable institutions are accustomed to issue. That the harrowing conditions which they describe do exist, there is rarely any doubt; but whether those who describe them are the best agencies through which to assist to relieve them is quite another question. It is one which individual members of the public are rarely in a position to answer in a satisfactory way for themselves, though a good deal of information may usually be gathered as to the working of any given institution from "Hospitals and Charities," as by its means it is possible to compare the work and arrangements of one institution with another. Obviously, however, no publication can take upon itself to condemn any institution *tantis verbis* except in extreme instances of abuse.

It is unfortunately true that some hospitals and charities exist which the public should steadily refuse to support. Some have been initiated in the past from motives which will not bear close scrutiny, and though many of these have died a natural death, others are still carried on. There is also a certain number of small institutions which are undesirable because the special treatment carried out in them would just as well form part of the general work of an ordinary hospital, and they are therefore superfluous. In some others, again, the management is hopelessly inefficient, or the arrangement of the buildings is so out of date and their financial affairs so utterly embarrassed, that any efforts of the managing body are bound to be futile, and a good deal of money wasted.

In any case abuses are liable to spring up in very small hospitals and charities carrying on their operations in large towns, because their relative importance is so minute that they excite the interest and observation of a very limited circle. Moreover, the expenses of administration in such establishments tend to form an excessive percentage of the total outlay. The running of a hospital is after all precisely comparable to that of any other business establishment, and the relative economy with which such businesses can be worked is in direct ratio to the magnitude of their operations: provided always that the "style" in which things are done is the same. In the case, however, of institutions for the healing of the sick, this qualification has no weight, for in them only one style is permissible and that is the best. Besides this, the building of any hospital anywhere is a very expensive matter, and as far as London is concerned, it may be said, broadly, that no quite small hospital can be properly constructed and properly equipped at a cost commensurate with the accommodation it provides and the scope of work which it is really able to undertake. Nor, as has to some extent been explained above, can as much be got out of them for the sum they cost in annual upkeep, as the same sum would effect if it were part of the expenditure of a large establishment.

It may be said generally, therefore, that very small hospitals in London are rarely to be encouraged, that the money spent in many of them on the machinery of matrons, dispensers, porters, and secretaries is wasted, and that it is a question whether it would not be better to allow all of them to die out. Certainly when there is a question of starting a fresh one or of removing or rebuilding an old one, its necessity at all should be placed beyond doubt, and the efficiency and skill of those who have taken upon themselves the management carefully considered.

Such matters individuals can rarely decide for themselves, and except in the case of well-known hospitals working in full view of numbers of competent critics, the majority of the public would be well advised to contribute what it can for hospital purposes either through the King's Fund or one of the other great hospital funds, and thus make perfectly certain that every farthing of its money will be well spent. Now that the League of Mercy is established the humblest donor can benefit the hospitals without difficulty or diffidence. The question is of importance because, although it is not true, as has been sometimes stated, that the average sum available for charities is practically definite and inelastic, yet there is no room whatever for waste. No doubt waste exists at all hospitals, especially those connected with medical schools. But where a great hospital of this type is directly controlled by a capable and experienced medical superintendent the public have the best possible guarantee of efficient administration. In selecting one of the larger hospitals as the recipient of a generous gift intending donors will be well advised to select by preference one which is administered under the direction of a resident medical superintendent.

